# Diffusing science through social networks: The case of breastfeeding communication on Twitter

**DOI:** 10.1371/journal.pone.0237471

**Published:** 2020-08-13

**Authors:** Sara Moukarzel, Martin Rehm, Miguel del Fresno, Alan J. Daly

**Affiliations:** 1 Larsson-Rosenquist Foundation Mother-Milk-Infant Center of Research Excellence, University of California San Diego, La Jolla, CA, United States of America; 2 Department of Education Studies, University of California San Diego, La Jolla, CA, United States of America; 3 Institute of Educational Consulting, University of Education Weingarten, Weingarten, Germany; 4 Department of Social Work, National Distance Education University, Madrid, Spain; Universitat de Barcelona, SPAIN

## Abstract

Breastfeeding is one of many health practices known to support the survival and health of mother and infant, yet low breastfeeding rates persist globally. These rates may be influenced by limited diffusion of evidence-based research and guidelines from the scientific community (SC). As recently highlighted by the National Academy of Sciences, there is a need for the SC to diffuse its findings to the public more effectively online, as means to counteract the spread of misinformation. In response to this call, we gathered data from Twitter for one month from major breastfeeding hashtags resulting in an interconnected social network (*n* = 3,798 users). We then identified 59 influencers who disproportionately influenced information flow using social network analysis. These influencers were from the SC (e.g. academics, researchers, health care practitioners), as well as interested citizens (IC) and companies. We then conducted an ego-network analysis of influencer networks, developed ego maps, and compared diffusion metrics across the SC, IC and company influencers. We also qualitatively analyzed their tweets (n = 711) to understand the type of information being diffused. SC influencers were the least efficient communicators. Although having the highest tweeting activity (80% of tweets), they did not reach more individuals compared to IC and companies (two-step ego size: 220± 99, 188 ± 124, 169 ± 97 respectively, *P* = 0.28). Content analysis of tweets suggest IC are more active than the SC in diffusing evidence-based breastfeeding knowledge, with 35% of their tweets around recent research findings compared to only 12% by the SC. Nonetheless, in terms of outreach to the general public, the two-step networks of SC influences were more heterogenous than ICs (55.7 ± 5.07, 50.9 ± 12.0, respectively, *P*<0.001). Collectively, these findings suggest SC influencers may possess latent potential to diffuse research and evidence- based practices. However, the research suggests specific ways to enhance diffusion.

## Introduction

Breastfeeding is one of many health practices which have been shown to support the survival and health of both mother and infant, and yet low breastfeeding rates persist around the world [1–3]. The World Health Organization (WHO) recommends infants be exclusively breastfed for the first six months of life followed by continued breastfeeding with food for two years or beyond [4]. If breastfeeding rates are increased to near-universal levels, the estimated death of 20,000 mothers and 800,000 children may be prevented annually [3]. Whether women follow breastfeeding guidelines depends on a complex range of sociocultural factors which include for example: how well parents and their social groups are informed about the research findings and skills related to breastfeeding; how positive a community’s attitude is towards breastfeeding; and how well supported parents are to initiate and continue breastfeeding within the health care system (e.g. access to trained staff), at the workplace (e.g. paid maternity leave), and in public (e.g. availability of breastfeeding rooms) [2,5–7].

From a public health standpoint, ensuring parents are well-informed and well-supported requires large-scale breastfeeding promotion efforts which strategically target members of society who can influence parents directly or indirectly through their words and actions (i.e., mother’s family and friends, providers of healthcare and childcare, employers and co-workers, policy makers) [8]. As shown in other health areas such chronic disease prevention, and more recently for breastfeeding promotion, social media may be a promising platform to execute such efforts at scale [9–11]. However, to adequately do so, theory-grounded analytic approaches are required to identify networks (groups of people who interact together), recognize influencers (leaders in these networks), and understand message diffusion pathways.

Within the breastfeeding promotion space, researchers have reported that social media channels such as Instagram and Facebook can support breastfeeding environments [11–13]. However, work in this area has typically only focused on content analysis of posts. What is missing in the breastfeeding literature is rigorous examination of breastfeeding networks, communities, influencers, and the diffusion of evidence-based knowledge. Our research group has recently begun to address this gap by studying the breastfeeding communication landscape on Twitter using social network theory (SNT) [14,15] and a host of analytic approaches we have used in other settings [16].

Social network theory (SNT) suggests that individuals are embedded in dense networks of social interactions and the pattern of these relations impacts the diffusion of opinions, communication, and ideas [16]. By analyzing these interactions, researchers can identify pathways to improve evidence-informed knowledge dissemination through networks of key influencers in online or offline spaces. We have recently shown that Twitter harbors a large interrelated breastfeeding social network (network of users exchanging information about breastfeeding) with unique sub-communities who shared a wide range of breastfeeding-related content [15]. The flow within this network was disproportionately influenced by a set of identifiable key influencers including: members of the *scientific community* (SC), *interested citizens* (IC), and *companies* [15]. Our analysis from this previous work indicated that the SC (e.g. academics, researchers, health care practitioners, non-governmental agencies) seem to have limited opportunities to disseminate evidence-based information to the lay public [15]. In this follow-up study using the same dataset, we take an innovative ego-net analysis approach to conduct a much deeper investigation into the extent to which information by the SC is diffused to the general public on Twitter as well as what type of information is being moved. Findings may help inform the development and implementation of a social network-focused intervention to improve evidence-informed knowledge diffusion on Twitter.

Ego-net analysis (ENA) is a less commonly-used approach to social network analysis [17]. An ego network represents the set of relational ties an ego forms with alters. In our case, *‘ego’* refers to an individual in the breastfeeding network and *alters* are others with whom they interact (i.e. tweet, retweet, reply, or mention). These relational ties reflect a “connection” through which social resources such as communication, opinions, and knowledge may flow [17]. Ego networks comprised of heterogeneous alters allow for the movement to and from ego of novel and non-redundant resources and in this sense are more “effective” at providing access to both opportunities to share and receive new relational resources (e.g. knowledge, approaches, ideas, etc.) [18–22]. When ego networks tend toward homophily, meaning alters are similar to ego on attributes such as background, beliefs, and knowledge, access to novel information is often limited and the diffusion of new perspectives is inhibited [23,24]. Homophilous networks are one barrier scientists face to effectively communicate their findings to a broader audience [25]. When it comes to informing the general public, heterogenous networks by the SC with large number of alters may provide a set of social ties that enable the SC to be more effective at diffusing resources.

While focus of this paper is on breastfeeding information diffusion, this work is under the umbrella of a much larger research agenda, outlined by the National Academies of Sciences, Engineering, and Medicine (NAS), which raises the need to better communicate science effectively [25]. Specifically, this study was conducted to better understand breastfeeding information diffusion by key influencers with an emphasis on the *scientific community* (SC) on Twitter by: 1) visualizing the ego networks of key influencers 2) determining and comparing the structural metrics of influencers’ ego networks among the SC, IC and companies, 3) determining and comparing the homophily/heterogeneity and composition of the networks and 4) describing and comparing the content being shared on Twitter by influencer category.

## Materials and methods

### a) Data collection

This is the second study in a series of analysis using data from Twitter. Using a dedicated server, we accessed the platform’s application programming interface (API), complying with the terms and conditions for Twitter [15]. More specifically, from December 18- January 18, 2020, we collected all tweets and user profile information from discussions that included at least one of the following hashtags: #breastfeed, #breastfeeding, #normalizebreastfeeding, #Breastfeeding, #breastmilk, #breastfeedingmoms and #breastfeedingsupport. Overall, we collected 3,972 Tweets from 1,993 unique users. On average, each of these users posted 4.05 Tweets (SD = 19.10, Median = 1.00, IQR = 1.00). Moreover, this group of unique users contacted an even larger group of others that did not actively partake in the hashtag conversations. Consequently, we eventually were able to collect user profiles from a total of 3,798 individuals. The tweets information consisted of tweet content, sender, type of message (tweet, mention or reply to) and date. The profile information included, among others, user details such as profile description, amount of followers and profile pictures.

### b) Data analysis

- **Social network analysis to identify key influencers.** A more detailed description of the methods used to identify influencers is provided, as recently published (S1 Appendix) [15]. Briefly, the overall network are constructed and users characterized by in-degree (number of times they were mentioned or retweeted), out-degree (number of tweets they sent) and overall degree centrality (both in- and out-going activity). Users who engaged in pornographic content around breastfeeding were removed (*n* = 1,324), then key influencers (*n* = 59) in the remaining network of 2,474 users were identified as the top 5% of users with highest overall degree centrality (S1 Table) [21].
- **Ego net analysis to describe influencer structural roles and characteristics.** In this paper, for each influencer identified earlier, two types of ego network were constructed using the igraph library of the statistical software R (S1 Fig). First, we created a “One-step” ego network describing each influencer’s immediate alters (users who retweet or mention the influencer) and the connections among these alters. This approach depicts and assesses the topology of an individual’s immediate network structure, and is described as an individual’s first “social circle”. Second, we created the “two-step” ego networks, which expand the analysis to include the alters of an influencer’s alters. By including these second “social circle” interactions, a more accurate calculation of diffusion of information can be done using more complex network metrics, such as indegree, outdegree, density, and homophily/heterogeneity [21,26]. As we are primarily interested in the ways in which the SC, such as researchers, diffuse information, we focus the results from the SC viewpoint.
- **Qualitative analysis of key influencers and their tweets.** Using inductive qualitative coding [27] and based on user profiles and tweeting history, we categorized influencers into three categories: *SC* (academics, researchers, health care practitioners, and/or non-governmental agencies), *IC* representing the general public, or *for-profit companies* [14]. Similarly, tweets within the aforementioned influencer categories were coded by two independent researchers who collaboratively developed a codebook based on actual discussions rather than *a priori*-set categories [27]. Differences in network metrics between the three influencer categories were analyzed using Kruskal Wallis test followed by Dunn- Bonferroni posthoc test for skewed data. For normally distributed data, ANOVA was used followed by Bonferroni posthoc test. Analyses were performed using SPSS (IBM SPSS Statistics for Mac, Version 26, Chicago, IL, USA). Level of significance was set at *p* values < 0.05.

## Results

### 1. Influencers from the scientific community diffuse information to the general public

**Fig 1:**
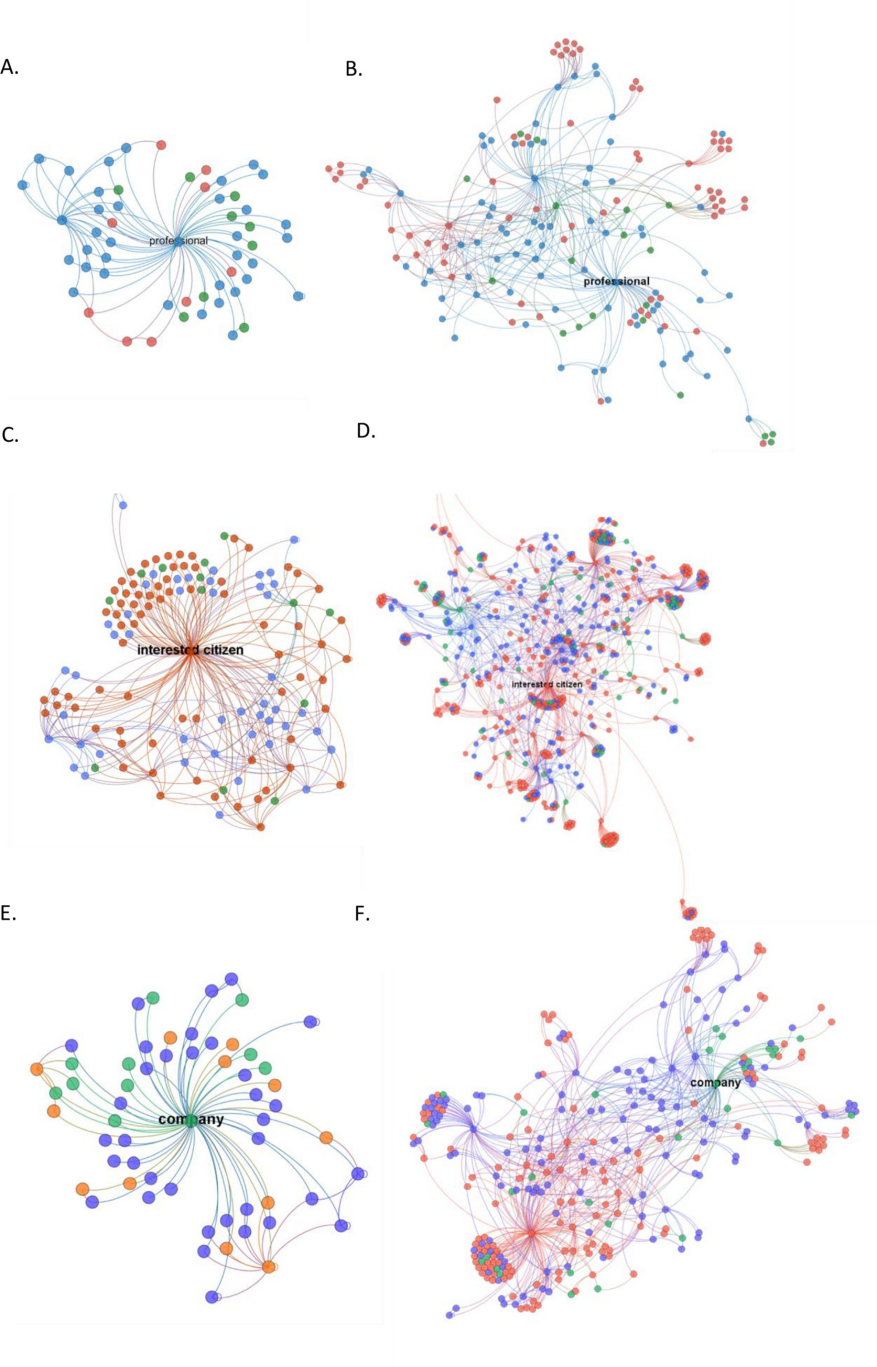
One-step and two-step ego network maps of the most influential influencers. Each dot represents a unique individual in the influencer’s breastfeeding network and the lines between the dots reflect exchanged tweets (tweets, retweets, or mentions). A and B represent the one-step and two-step ego network maps respectively of the most influential member of the SC; C and D represent those of the most influential interested citizen; E and F represent those of the most influential company. Blue, users from the scientific community; Red, users who are interested citizens; Green: companies.

Fig 1 presents a visualization of the ego networks of the three most influential users within the SC, IC, and company categories respectively. The figure provides exemplars of the “one-step” and “two-step” sociograms (ego networks) constructed for all 59 key influencers. As a starting point of analysis, these sociograms indicate several relevant findings. First, it seems influencers from the SC are not only diffusing information to others in their community, but also to IC. Second, IC themselves seem to be diffusing information generated from the SC to others in the general public, as well as creating and diffusing their own content. Third, while companies seem to be engaged in diffusing information from the SC, their most pronounced online activity is diffusing company-related content to the general public.

### 2. Influencers from the scientific community are at a disadvantage when it comes to the number of users reached

No significant differences in the structural ego network metrics were found across influencer categories (Table 1). The mean ego size of SC’s network, reflecting the number of users reached, was 22 users at one-step and increased to 221 at two-step. This level of connectivity was not significantly different from the IC and companies. We also measured the *density* of the ego networks at step one and step two and results indicate that the SC had a density of 10% of users at one-step and 1% at two-step, which was consistent with density measures for other influencer networks.

**Table 1:** Differences in structural metrics of key influencers’ ego networks by category.

		Key Influencer		*P*^2^
	SC influencers *n* = 26	IC influencers *n* = 30	Influencer as a company *n* = 7	
**General**^1^				
Degree	14 (22)	16 (14)	13 (45)	0.82
Indegree	9 (13)	0 (12)	12 (12)	0.20
Outdegree	1 (17)	10 (22)	0 (56)	0.46
**One-Step**^2^				
Ego size	22 ± 15	25 ± 21	27 ± 22	0.79
Density	0.10 ± 0.06	0.10 ± 0.06	0.07 ± 0.04	0.54
**Two-Step**^2^				
Ego size	221 ± 99	188 ± 124	169 ± 97	0.28
Density	0.01 ± 0.01	0.02 ± 0.01	0.02 ±0.01	0.23

SC, scientific community. IC, interested citizen.

^1^Values are median (IQR) for skewed data and differences determined by Kruskal Wallis Test.

^2^Values are mean ± SD for normally distributed data and differences determined by ANOVA followed by Bonferroni Posthoc test. Values with different letters are significantly different.

### 3. Influencers from the scientific community had less heterogenous relations than companies

Perhaps due to brand competition and the nature of for-profit businesses, company networks were the most heterogenous: 81–85% of users in companies’ networks were non-company users at one-step and two-step (Table 2). Interestingly, while influencers in the SC did not reach more users (as reflected by their networks’ ego size and density), their networks were significantly more heterogenous at two-step than IC. Conversely, there are less members from the SC in the SC influencer networks at two-step (44%) compared to interested citizens in the IC influencer networks (49%). Collectively, these findings suggest that influencers from the SC may possess latent potential to diffuse information to a broader audience and an opportunity to have even greater heterogenous networks similar to the profile of companies.

**Table 2:** Differences in heterogeneity and network distribution by category.

	Key Influencer Category			*P-Value*^2^
	SC influencers *n* = 26	IC influencers *n* = 30	Company influencers *n* = 7	
**One-step**				
Heterogeneity Network distribution,%	61.2 ± 21.7^a^	51 ± 23.5^a^	80.6 ± 15.0^b^	0.006
Member of the SC	38.8 ± 21.7	29.4 ± 21.8	26.1 ± 23.5	0.245
IC	43.1 ± 18.8	49.0 ± 23.5	54.6 ± 33.5	0.603
Companies	18.1 ± 16.2	21.6 ± 23.0^1^	19.4 ± 15.0	0.773
**Two-step**				
Heterogeneity Network distribution,%	55.7 ± 5.07^a^	50.9 ± 12.0^b^	85.3 ± 3.03^c^	<0.001
Members of the SC	44.3 ± 5.07^a^	39.2 ± 9.89^b^	39.5 ± 11.6^a,b^	0.008
IC	41.2 ± 8.39^a^	49.1 ± 12.0^b^		0.008
Companies	14.5 ± 4.84^a^	11.7 ± 5.05^b^		0.042

SC, scientific community. IC, interested citizen. Values are mean ± SD. In overall network: SC, n = 769; IC, n = 1350; Company, n = 355.

^1^ skewed with median = 11.3 and IQR = 14.2.

^2^Differences determined by Kruskal Wallis Test. Values with different letters are significantly different, determined by Dunn-Bonferroni Posthoc test.

### 4. Influencers from the scientific community engage in more activity, but reach fewer unique individuals

To better understand the type of information being diffused in influencers’ networks, the content analysis of their tweets was reported by category (Table 3). First, tweets from the SC were the most common, accounting for approximately 80% of total influencers’ tweets, although the SC had similar or smaller networks. Taken together, it seems the SC, while active, is less efficient in reaching a wider audience as compared to IC and companies.

**Table 3:** Content analysis of tweets by influencer category (*n* = 711 tweets).

	SC	IC	Companies
Number of tweets (%)	493 (81.2)	95 (15.7)	19 (3.13)
Identified themes, %			
Commercial Use	41.6	2.1	36.8
Professional Communication & Invitations	23.5	0.0	0.0
Research Findings & evidence-based recommendations	11.8	34.7	5.3
Breastfeeding Advocacy	6.3	16.8	21.1
Policy Awareness	5.9	0.0	0.0
Non-evidence based Recommendations	5.7	9.5	15.8
Community Engagement	3.0	27.4	0.0
Personal Anecdotes	2.2	0.0	21.1
Other (beauty, veganism)	0.0	9.5	0.0

SC, influencers from the scientific community. IC, influencers who are interested citizens.

### 5. Influencers from the scientific community primarily use networks for research, announcements, and commercial purposes

The highest percentage of tweets by both the SC and companies was for commercial purposes (e.g, sales of books or dietary supplements) not for sharing research findings or for more direct non-profit breastfeeding promotion and support (Table 3). In addition, the SC commonly used Twitter to share details about research talks and clinical training events they were giving (24% of tweets), as well as to share findings from recently published peer-reviewed articles (12%).

Interestingly, IC were more likely to share research findings (35%), followed by community engagement such as encouraging parents to join breastfeeding support groups and then breastfeeding advocacy such as encouraging public breastfeeding. This suggests that IC were actually far more active than the SC in diffusing evidence-based knowledge around breastfeeding, which may seem counter-intuitive. Companies on the other hand, which seem more efficient in diffusing information into their networks, tended to share more non-evidence based recommendations than research findings.

## Discussion

This study was conducted to expand understanding around whether, by whom, and how efficiently and effectively evidence-based breastfeeding information is diffused on Twitter based on the analysis of influencer ego networks with a focus on the SC. Our findings suggest Twitter is an opportunity space for the scientific community including researchers to effectively communicate science to the public. However, the SC does face a wide range of identified challenges that can be addressed to ensure wide diffusion of accurate information.

We found that influencers with academic and/or clinical credentials are highly active in sharing research findings and clinical recommendations about breastfeeding on Twitter. However, despite their relatively high number of tweets (498 tweets in one month), these influencers do not have wide public outreach (reaching around 91 lay individuals only). On the other hand, influencers who are IC tweet much less frequently about breastfeeding (95 tweets/month), yet create the same level of user engagement as the SC (reaching around 92 lay individuals as well). To put things into perspective, if the SC influencers were as efficient as IC, their outreach would increase five-folds by engaging 482 and not 91 lay individuals over the same study period.

The solution is not only to simply create, organically or concertedly, larger and more heterogeneous influencer networks, but also to be aware of the risk of inaccurate knowledge diffusion by lay influencers and companies. Health misinformation on social media are known to diffuse much “farther, faster, and deeper” than scientifically-sound information [28]. Also, individuals who use social media to discuss science-related controversies often disproportionately have views against the scientific consensus [29]. While we found no clear evidence of such activity by influencers in our study, our qualitative methods were not designed to detect subtle nuances in knowledge translation activity by all lay individuals in the network. It is possible that findings gleaned and shared by the public may be inaccurately reported or taken out of context, unintentionally leading to a spread of misinformation and non-evidence based practices. Future studies need to address whether evidence-based information diffused by lay influencers are “lost in translation”.

The need to improve the SC’s outreach online as means to increase diffusion of scientific information to the public is not an issue only within the breastfeeding field. Rather, the disconnect between generating findings and communicating those findings to interested individuals extends to a wide range of fields within medicine and engineering, as recently discussed in the research agenda report by NAS [25]. The NAS report notes that understanding social networks and the diffusion of knowledge through social networks is one of the key issues related to the diffusion of evidence-based practices vexing multiple disciplines. Similarly, there is increased interest in the field of altmetrics to develop methods for identifying and measuring the societal impacts of research, one of which is social network analysis [30]. Recent advances in the field of altmetrics include identifying Facebook as an important yet underestimated medium for science and scholarly communication [31,32]. Additionally, analyses of user profiles as well as Facebook and Twitter post content and number of posts, show that non-academics do post and share links to research articles, and a diffusion of knowledge from scholars to the general public does exist in a wide range of academic disciplines [33–35]. However, the nuanced study of communication pathways among and across scholars, the public, and for-profit corporations seems to be limited. As such, this case study focusing on breastfeeding information shows how using social network analysis can further enrich the existing altmetrics literature and provide both visual and analytical data on communication pathways.

In a real sense, the SC related to breastfeeding and other disciplines operate in a networked society, and the ability to diffuse science effectively will require a host of new skills and proficiency in *social network literacy*. Despite the fact that we live in a hyper-connected socially networked world, researchers do not always systematically and explicitly focus on the social network literacy skills necessary to leverage their networks. Understanding how to connect to and leverage this larger social infrastructure as we identify in this paper may be critical in moving messages, accessing information, determining veracity, supporting decision-making, and connecting with others for discovery, community, and sharing of viewpoints [16]. An expanded exploration of why some lay individuals and companies have more efficient outreach cannot be answered within the scope of this paper. However, identifying the reasons might help provide the research community, within and beyond the breastfeeding field, with strategies to improve outreach. Our work raises several important research questions that may push the work forward: Compared to members of the SC, do IC attract a larger number of engaged users? Is the language used by IC more accessible to a lay audience (e.g. less use of jargon)? Is the tone of messages and sentiments by IC more interesting and engaging?

Given the fact that IC are reaching such a large group, another innovative strategy to improve effective science communication may be to build the capacity of the IC themselves in helping spread evidence in a coherent and accurate manner which reflects actual findings. Our data shows that influencers who are IC are efficient communicators. By engaging these identifiable influencers, it may be possible to augment the accuracy of their messages and leverage their active networks as complementary means of disseminating science. So while important to support the SC in better diffusing evidence, a supplemental strategy is to leverage the existing networks of IC in diffusing evidence-based findings and practices.

To our knowledge, this is the first study to use an ego network analytic approach to map the landscape of breastfeeding communication online. This work extends other face to face network research around the role of social influence on breastfeeding knowledge and attitudes. For example, we have shown that the composition of medical trainees’ social networks explain the extent of their knowledge and efficacy to support breastfeeding [36,37]. Collectively, these findings suggest that we may be better able to improve evidence-informed knowledge diffusion by studying networks of key influencers in both face to face and virtual spaces. Instead of solely identifying influencers based on number of followers on social media, our work expands this idea to suggest that influence is also a matter of social structural position in a network (e.g. being more central), as suggested in social network theory-grounded research [17].

The low number of tweets data (4,000 tweets) and the short study duration (one month) are two limitations of this study. There is a need for future longer studies to collect and analyze a larger sample, especially for users from the SC and companies to confirm or refute our findings particularly as they related to heterogeneity of influencer ego networks. Now that this research has identified influencers in the breastfeeding-focused scientific community and has shown their connectedness with the general public directly and through several influencers from the public itself, future research may include a social network-focused intervention to improve evidence-informed knowledge diffusion. By making influencers aware of their social structural position in the breastfeeding network, connecting influencers from the scientific community with those who are interested citizens, educating both of them on strategies to widen their outreach and improve accuracy of messaging, it may be possible to improve evidence-based knowledge diffusion on Twitter. Lessons learned in the breastfeeding space may be extended to other research fields.

## Supporting information

S1 Appendix Social network analytical approach to identify influencers. (DOCX)

S1 Table Description of identified key influencers. HCP, health care practitioner; NGO, non-governmental organization; BF, breastfeeding. *n* = 59 influencers.
(DOCX)

S1 Fig Illustration of a one-step and two-step ego network map. One-step, describes an influencer’s (ego’s) immediate alters (users who retweet or mention the influencer) and the connections among these alters. Two-step, describes an influencer’s one-step network as well as the alters of the alters.
(DOCX)
