# Supplementary material for: Diffusing science through social networks: The case of breastfeeding communication on Twitter
Source: PLoS One. 2020 Aug 13;15(8):e0237471. doi: 10.1371/journal.pone.0237471 (PMC7425887; doi:10.1371/journal.pone.0237471)
Supplement: S1 Appendix — (DOCX) [file pone.0237471.s001.docx]

**S1. Appendix**

We started with a social network analyses (SNA) to identify the overall network and underlying communication patterns. First, we constructed the overall social network around breastfeeding and then computed the following centrality metrics: in-degree (incoming tweets, e.g. being mentioned or retweeted), out-degree (outgoing tweets, e.g. sending a tweet), and overall degree centrality (both in- and out-going activity). These metrics provided an indication of how often an individual has been contacted or has contacted others, respectively. We also determined underlying community structures using the Louvain method (De Nooy, Mrvar, & Batagelj, 2011).

We then identified key influencers with prominent social structural roles (top 5% of individuals based on overall degree centrality) in the network, not on traditional metrics of number of followers as we have done in our other work. Given the social structural position of these individuals, they are regarded as having disproportionate influence over content, information, and ideas that flow in the breastfeeding network. (Del Fresno et al., 2016).

References:

De Nooy W, Andrej M, Vladimir B. Exploratory social network analysis with Pajek. Cambridge, UK: Cambridge University Press; 2011.

Del Fresno M, Daly AJ, Segado Sánchez-Cabezudo S. Identifying the new Influencers in the Internet Era: Social Media and Social Network Analysis. *Revista Española de Investigaciones Sociológicas, 2016;*153: 121-140
