# Supplementary material for: Diffusing science through social networks: The case of breastfeeding communication on Twitter
Source: PLoS One. 2020 Aug 13;15(8):e0237471. doi: 10.1371/journal.pone.0237471 (PMC7425887; doi:10.1371/journal.pone.0237471)
Supplement: S1 Table — HCP, health care practitioner; NGO, non-governmental organization; BF, breastfeeding. n = 59 influencers. (DOCX) [file pone.0237471.s002.docx]

**S1 Table 1.**

| Description of key influencers | n (%) |
| --- | --- |
| Professionals  Researcher  Researcher & HCP  HCP  Academic journal  NGO | 28 (47.4)  4 (6.8)  4 (6.8)  9 (15.3)  2 (3.4)  9 (15.3) |
| Interested citizens  Support BF  Does not support BF | 24 (40.7)  14 (23.7)  10 (17.0) |
| Companies | 7 (11.9) |
