# Supplementary figures and images for: Diffusing science through social networks: The case of breastfeeding communication on Twitter
Source: PLoS One. 2020 Aug 13;15(8):e0237471. doi: 10.1371/journal.pone.0237471 (PMC7425887; doi:10.1371/journal.pone.0237471)

**S1 Figure**


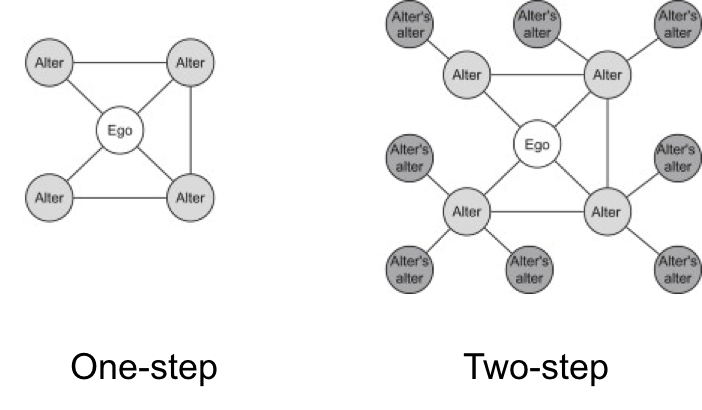

Supplement: S1 Fig — One-step, describes an influencer’s (ego’s) immediate alters (users who retweet or mention the influencer) and the connections among these alters. Two-step, describes an influencer’s one-step network as well as the alters of the alters. (DOCX) [file pone.0237471.s003.docx]
